# Ethyl 6-methyl-2-*p*-tolyl­pyrazolo[1,5-*a*]pyridine-5-carboxyl­ate

**DOI:** 10.1107/S1600536809035314

**Published:** 2009-09-09

**Authors:** Jiong Jia, Yan-qing Ge, Gui-long Zhao, Jian-wu Wang

**Affiliations:** a School of Chemistry and Chemical Engineering, Shandong University, Jinan 250100, People’s Republic of China

## Abstract

In the title mol­ecule, C_18_H_18_N_2_O_2_, the bicyclic ring system and the benzene ring form a dihedral angle of 13.45 (3)°. In the crystal structure, weak inter­molecular C—H⋯O hydrogen bonds link mol­ecules into chains propagated along [201].

## Related literature

For novel pyrazolo[1,5-*a*]pyridine compounds, see: Ge *et al.* (2009 ▶). For a related structure, see: Shao *et al.* (2009 ▶).
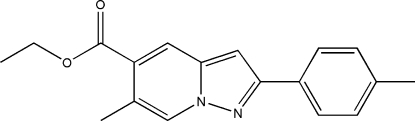


## Experimental

### 

#### Crystal data

C_18_H_18_N_2_O_2_
*M* *_r_* = 294.34
Monoclinic, 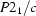

*a* = 6.8352 (3) Å
*b* = 30.3999 (11) Å
*c* = 7.5409 (3) Å
β = 97.375 (2)°
*V* = 1553.96 (11) Å^3^
*Z* = 4
Mo *K*α radiation
μ = 0.08 mm^−1^
*T* = 293 K
0.43 × 0.32 × 0.21 mm

#### Data collection

Bruker SMART CCD area-detector diffractometer
Absorption correction: multi-scan (*SADABS*; Sheldrick, 1996 ▶) *T*_min_ = 0.965, *T*_max_ = 0.983
18651 measured reflections
3181 independent reflections
2166 reflections with *I* > 2σ(*I*)
*R*_int_ = 0.036

#### Refinement

*R*[*F* ^2^ > 2σ(*F* ^2^)] = 0.056
*wR*(*F* ^2^) = 0.148
*S* = 1.07
3181 reflections
199 parameters
H-atom parameters constrained
Δρ_max_ = 0.23 e Å^−3^
Δρ_min_ = −0.22 e Å^−3^

### 

Data collection: *SMART* (Bruker, 1998 ▶); cell refinement: *SAINT* (Bruker, 1999 ▶); data reduction: *SAINT*; program(s) used to solve structure: *SHELXTL* (Sheldrick, 2008 ▶); program(s) used to refine structure: *SHELXTL*; molecular graphics: *SHELXTL*; software used to prepare material for publication: *SHELXTL*.

## Supplementary Material

Crystal structure: contains datablocks I, global. DOI: 10.1107/S1600536809035314/cv2611sup1.cif

Structure factors: contains datablocks I. DOI: 10.1107/S1600536809035314/cv2611Isup2.hkl

Additional supplementary materials:  crystallographic information; 3D view; checkCIF report

## Figures and Tables

**Table 1:** Hydrogen-bond geometry (Å, °)

*D*—H⋯*A*	*D*—H	H⋯*A*	*D*⋯*A*	*D*—H⋯*A*
C9—H9*A*⋯O3^i^	0.93	2.42	3.339 (3)	170

Symmetry code: (i) 
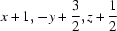
.

**Fig. 1.**
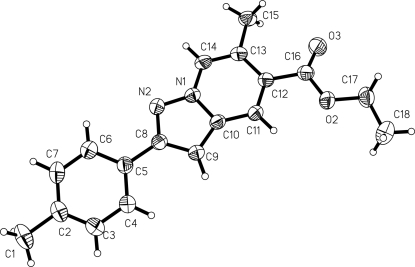
View of the title compound, with displacement ellipsoids drawn at the 40% probability level.

## supplementary crystallographic information

### Comment

The pyrazolo[1,5-*a*]pyridine derivatives have been of interest for their pharmacological and biological activities. Considerable efforts of our group have been devoted to the development of novel pyrazolo[1,5-*a*]pyridine compounds(Ge *et al.*, 2009). In continuation of this work, we report here the crystal structure of the title compound, (I) (Fig. 1).

In (I), all bond lengths are normal and in a good agreement with those reported previously (Shao *et al.*, 2009). Atoms O2/O3/C15/C16/C17/C18 lie in 1*H*-pyrazolo[1,5-*a*]pyridine (C8—C14/N1/N2) plane with the maximum deviation of 0.065 (3) Å for O2. The 1*H*-pyrazolo[1,5-*a*]pyridine plane forms dihedral angle of 13.45 (3)° with the benzene ring (C2—C7).

In the crystal structure, weak intermolecular C–H···O hydrogen bond (Table 1) link the molecules into chains propagated in direction [201].

### Experimental

To a 50-ml round-bottomed flask were added 3-*p*-tolyl-1*H*-pyrazole-5-carbaldehyde(6.0 mmol), ethyl 4-bromo-3-methylbut-2-enoate (7.2 mmol), potassium carbonate (1.60 g, 12.5 mmol) and DMF (10 mL). The mixture was stirred at rt for 8 h and then filtered. The filtrate was poured into water (100 ml) and extracted with CH_2_Cl_2_ (3 *x* 30 ml). The combined extracts were washed with water (2 *x* 50 ml), dried over anhydrous MgSO4 and filtered, and the solvent was removed by rotary evaporation. The crude product was purified by column chromatography (yield 75%). Crystals of (I) suitable for X-ray diffraction were obtained by slow cooling of the refluxed solution of the product in ethyl acetate at room temperature for 2 d.

### Refinement

All H atoms were placed in calculated positions [C–H = 0.93–0.97 Å], and included in the final cycles of refinement using a riding model, with *U*_iso_(H) = 1.2*U*_eq_(C) and 1.5*U*_eq_(C) for the methyl H atoms.

### Crystal data

**Table:**

C_18_H_18_N_2_O_2_	*F*(000) = 624
*M**_r_* = 294.34	*D*_x_ = 1.258 Mg m^−^^3^
Monoclinic, *P*2_1_/*c*	Mo *K*α radiation, λ = 0.71073 Å
Hall symbol: -P 2ybc	Cell parameters from 4574 reflections
*a* = 6.8352 (3) Å	θ = 2.7–24.2°
*b* = 30.3999 (11) Å	µ = 0.08 mm^−^^1^
*c* = 7.5409 (3) Å	*T* = 293 K
β = 97.375 (2)°	Block, colourless
*V* = 1553.96 (11) Å^3^	0.43 × 0.32 × 0.21 mm
*Z* = 4	

### Data collection

**Table:**

Bruker SMART CCD area-detector diffractometer	3181 independent reflections
Radiation source: fine-focus sealed tube	2166 reflections with *I* > 2σ(*I*)
graphite	*R*_int_ = 0.036
φ and ω scans	θ_max_ = 26.3°, θ_min_ = 1.3°
Absorption correction: multi-scan (*SADABS*; Sheldrick, 1996)	*h* = −8→8
*T*_min_ = 0.965, *T*_max_ = 0.983	*k* = −37→37
18651 measured reflections	*l* = −9→9

### Refinement

**Table:**

Refinement on *F*^2^	Primary atom site location: structure-invariant direct methods
Least-squares matrix: full	Secondary atom site location: difference Fourier map
*R*[*F*^2^ > 2σ(*F*^2^)] = 0.056	Hydrogen site location: inferred from neighbouring sites
*wR*(*F*^2^) = 0.148	H-atom parameters constrained
*S* = 1.07	*w* = 1/[σ^2^(*F*_o_^2^) + (0.0589*P*)^2^ + 0.5452*P*] where *P* = (*F*_o_^2^ + 2*F*_c_^2^)/3
3181 reflections	(Δ/σ)_max_ < 0.001
199 parameters	Δρ_max_ = 0.23 e Å^−^^3^
0 restraints	Δρ_min_ = −0.22 e Å^−^^3^

### Special details

**Table:**

Geometry. All e.s.d.'s (except the e.s.d. in the dihedral angle between two l.s. planes) are estimated using the full covariance matrix. The cell e.s.d.'s are taken into account individually in the estimation of e.s.d.'s in distances, angles and torsion angles; correlations between e.s.d.'s in cell parameters are only used when they are defined by crystal symmetry. An approximate (isotropic) treatment of cell e.s.d.'s is used for estimating e.s.d.'s involving l.s. planes.
Refinement. Refinement of *F*^2^ against ALL reflections. The weighted *R*-factor *wR* and goodness of fit *S* are based on *F*^2^, conventional *R*-factors *R* are based on *F*, with *F* set to zero for negative *F*^2^. The threshold expression of *F*^2^ > σ(*F*^2^) is used only for calculating *R*-factors(gt) *etc*. and is not relevant to the choice of reflections for refinement. *R*-factors based on *F*^2^ are statistically about twice as large as those based on *F*, and *R*- factors based on ALL data will be even larger.

### Fractional atomic coordinates and isotropic or equivalent isotropic displacement parameters (Å^2^)

**Table:**

	*x*	*y*	*z*	*U*_iso_*/*U*_eq_	
N1	0.3372 (2)	0.65607 (5)	0.5370 (2)	0.0429 (4)	
N2	0.3988 (2)	0.61395 (6)	0.5589 (2)	0.0482 (4)	
O2	0.3651 (2)	0.81645 (4)	0.5569 (2)	0.0561 (4)	
O3	0.0666 (3)	0.80858 (6)	0.4073 (3)	0.0806 (6)	
C1	1.0771 (5)	0.46224 (8)	0.7690 (4)	0.0820 (8)	
H1A	1.2123	0.4711	0.7975	0.123*	
H1B	1.0627	0.4450	0.6615	0.123*	
H1C	1.0386	0.4450	0.8654	0.123*	
C2	0.9475 (4)	0.50258 (8)	0.7418 (3)	0.0589 (6)	
C3	1.0264 (4)	0.54415 (8)	0.7553 (3)	0.0638 (6)	
H3A	1.1619	0.5474	0.7852	0.077*	
C4	0.9101 (3)	0.58130 (7)	0.7255 (3)	0.0563 (6)	
H4A	0.9683	0.6090	0.7366	0.068*	
C5	0.7080 (3)	0.57786 (6)	0.6794 (3)	0.0460 (5)	
C6	0.6282 (4)	0.53587 (7)	0.6683 (3)	0.0636 (6)	
H6A	0.4927	0.5324	0.6392	0.076*	
C7	0.7463 (4)	0.49920 (8)	0.6995 (4)	0.0695 (7)	
H7A	0.6885	0.4715	0.6918	0.083*	
C8	0.5871 (3)	0.61754 (6)	0.6386 (3)	0.0435 (5)	
C9	0.6428 (3)	0.66109 (7)	0.6684 (3)	0.0464 (5)	
H9A	0.7643	0.6714	0.7221	0.056*	
C10	0.4813 (3)	0.68603 (6)	0.6023 (3)	0.0423 (5)	
C11	0.4335 (3)	0.73089 (6)	0.5842 (3)	0.0440 (5)	
H11A	0.5260	0.7519	0.6288	0.053*	
C12	0.2535 (3)	0.74423 (7)	0.5024 (2)	0.0412 (5)	
C13	0.1075 (3)	0.71204 (7)	0.4353 (3)	0.0441 (5)	
C14	0.1553 (3)	0.66890 (7)	0.4564 (3)	0.0473 (5)	
H14A	0.0628	0.6476	0.4153	0.057*	
C15	−0.0942 (3)	0.72349 (8)	0.3412 (3)	0.0557 (6)	
H15A	−0.1660	0.6970	0.3083	0.084*	
H15B	−0.0807	0.7403	0.2357	0.084*	
H15C	−0.1644	0.7405	0.4197	0.084*	
C16	0.2140 (3)	0.79236 (7)	0.4827 (3)	0.0476 (5)	
C17	0.3473 (4)	0.86376 (7)	0.5387 (3)	0.0624 (6)	
H17A	0.2408	0.8744	0.6007	0.075*	
H17B	0.3190	0.8718	0.4135	0.075*	
C18	0.5387 (4)	0.88348 (8)	0.6182 (3)	0.0757 (8)	
H18A	0.5313	0.9149	0.6078	0.114*	
H18B	0.6429	0.8727	0.5557	0.114*	
H18C	0.5649	0.8755	0.7421	0.114*	

### Atomic displacement parameters (Å^2^)

**Table:**

	*U*^11^	*U*^22^	*U*^33^	*U*^12^	*U*^13^	*U*^23^
N1	0.0375 (9)	0.0432 (10)	0.0472 (9)	−0.0043 (7)	0.0016 (7)	−0.0009 (7)
N2	0.0473 (10)	0.0404 (10)	0.0560 (10)	−0.0027 (8)	0.0035 (8)	−0.0002 (8)
O2	0.0546 (9)	0.0407 (9)	0.0710 (10)	−0.0009 (7)	0.0008 (7)	0.0056 (7)
O3	0.0562 (10)	0.0558 (11)	0.1210 (15)	0.0076 (8)	−0.0225 (10)	0.0140 (10)
C1	0.101 (2)	0.0602 (17)	0.0863 (19)	0.0314 (15)	0.0167 (16)	0.0084 (14)
C2	0.0711 (17)	0.0490 (14)	0.0574 (13)	0.0134 (12)	0.0116 (11)	0.0033 (10)
C3	0.0520 (14)	0.0598 (16)	0.0790 (17)	0.0093 (12)	0.0058 (12)	0.0061 (12)
C4	0.0516 (13)	0.0448 (13)	0.0719 (15)	0.0004 (10)	0.0062 (11)	0.0037 (11)
C5	0.0476 (12)	0.0423 (12)	0.0487 (11)	0.0007 (9)	0.0078 (9)	−0.0003 (9)
C6	0.0553 (14)	0.0488 (14)	0.0850 (17)	−0.0039 (11)	0.0027 (12)	−0.0019 (12)
C7	0.0785 (18)	0.0384 (13)	0.0909 (19)	−0.0012 (12)	0.0081 (14)	0.0013 (12)
C8	0.0414 (11)	0.0436 (12)	0.0454 (11)	−0.0024 (9)	0.0050 (9)	0.0006 (9)
C9	0.0388 (11)	0.0441 (12)	0.0547 (12)	−0.0031 (9)	−0.0007 (9)	0.0001 (9)
C10	0.0372 (10)	0.0432 (12)	0.0455 (11)	−0.0049 (9)	0.0022 (8)	−0.0004 (8)
C11	0.0405 (11)	0.0407 (11)	0.0500 (11)	−0.0051 (9)	0.0022 (9)	−0.0001 (9)
C12	0.0374 (10)	0.0451 (12)	0.0411 (10)	−0.0001 (9)	0.0045 (8)	0.0038 (8)
C13	0.0361 (10)	0.0534 (13)	0.0425 (11)	−0.0011 (9)	0.0036 (8)	0.0030 (9)
C14	0.0367 (11)	0.0533 (13)	0.0502 (12)	−0.0071 (9)	−0.0008 (9)	−0.0013 (9)
C15	0.0408 (12)	0.0626 (14)	0.0609 (14)	−0.0007 (10)	−0.0042 (10)	0.0036 (11)
C16	0.0406 (11)	0.0510 (13)	0.0512 (12)	0.0006 (10)	0.0055 (9)	0.0055 (10)
C17	0.0741 (17)	0.0418 (13)	0.0731 (16)	0.0030 (12)	0.0157 (13)	0.0056 (11)
C18	0.094 (2)	0.0588 (16)	0.0740 (17)	−0.0194 (14)	0.0101 (15)	−0.0054 (13)

### Geometric parameters (Å, °)

**Table:**

N1—N2	1.351 (2)	C7—H7A	0.9300
N1—C14	1.369 (2)	C8—C9	1.388 (3)
N1—C10	1.385 (2)	C9—C10	1.379 (3)
N2—C8	1.353 (2)	C9—H9A	0.9300
O2—C16	1.330 (2)	C10—C11	1.405 (3)
O2—C17	1.448 (3)	C11—C12	1.365 (3)
O3—C16	1.198 (2)	C11—H11A	0.9300
C1—C2	1.511 (3)	C12—C13	1.442 (3)
C1—H1A	0.9600	C12—C16	1.492 (3)
C1—H1B	0.9600	C13—C14	1.356 (3)
C1—H1C	0.9600	C13—C15	1.508 (3)
C2—C3	1.373 (3)	C14—H14A	0.9300
C2—C7	1.375 (4)	C15—H15A	0.9600
C3—C4	1.383 (3)	C15—H15B	0.9600
C3—H3A	0.9300	C15—H15C	0.9600
C4—C5	1.384 (3)	C17—C18	1.493 (3)
C4—H4A	0.9300	C17—H17A	0.9700
C5—C6	1.386 (3)	C17—H17B	0.9700
C5—C8	1.472 (3)	C18—H18A	0.9600
C6—C7	1.379 (3)	C18—H18B	0.9600
C6—H6A	0.9300	C18—H18C	0.9600
			
N2—N1—C14	125.13 (17)	C9—C10—C11	137.25 (19)
N2—N1—C10	112.56 (16)	N1—C10—C11	117.27 (17)
C14—N1—C10	122.30 (17)	C12—C11—C10	121.13 (18)
N1—N2—C8	103.98 (15)	C12—C11—H11A	119.4
C16—O2—C17	117.10 (17)	C10—C11—H11A	119.4
C2—C1—H1A	109.5	C11—C12—C13	120.00 (19)
C2—C1—H1B	109.5	C11—C12—C16	118.51 (18)
H1A—C1—H1B	109.5	C13—C12—C16	121.49 (17)
C2—C1—H1C	109.5	C14—C13—C12	117.99 (18)
H1A—C1—H1C	109.5	C14—C13—C15	118.08 (18)
H1B—C1—H1C	109.5	C12—C13—C15	123.93 (19)
C3—C2—C7	117.3 (2)	C13—C14—N1	121.30 (19)
C3—C2—C1	121.3 (2)	C13—C14—H14A	119.3
C7—C2—C1	121.5 (2)	N1—C14—H14A	119.3
C2—C3—C4	121.8 (2)	C13—C15—H15A	109.5
C2—C3—H3A	119.1	C13—C15—H15B	109.5
C4—C3—H3A	119.1	H15A—C15—H15B	109.5
C3—C4—C5	120.9 (2)	C13—C15—H15C	109.5
C3—C4—H4A	119.6	H15A—C15—H15C	109.5
C5—C4—H4A	119.6	H15B—C15—H15C	109.5
C4—C5—C6	117.2 (2)	O3—C16—O2	122.3 (2)
C4—C5—C8	120.38 (19)	O3—C16—C12	125.6 (2)
C6—C5—C8	122.4 (2)	O2—C16—C12	112.17 (17)
C7—C6—C5	121.1 (2)	O2—C17—C18	107.6 (2)
C7—C6—H6A	119.4	O2—C17—H17A	110.2
C5—C6—H6A	119.4	C18—C17—H17A	110.2
C2—C7—C6	121.7 (2)	O2—C17—H17B	110.2
C2—C7—H7A	119.2	C18—C17—H17B	110.2
C6—C7—H7A	119.2	H17A—C17—H17B	108.5
N2—C8—C9	111.98 (17)	C17—C18—H18A	109.5
N2—C8—C5	120.22 (18)	C17—C18—H18B	109.5
C9—C8—C5	127.78 (18)	H18A—C18—H18B	109.5
C10—C9—C8	106.01 (17)	C17—C18—H18C	109.5
C10—C9—H9A	127.0	H18A—C18—H18C	109.5
C8—C9—H9A	127.0	H18B—C18—H18C	109.5
C9—C10—N1	105.47 (17)		
			
C14—N1—N2—C8	178.09 (17)	C14—N1—C10—C9	−178.47 (17)
C10—N1—N2—C8	−0.5 (2)	N2—N1—C10—C11	179.20 (16)
C7—C2—C3—C4	−0.8 (4)	C14—N1—C10—C11	0.5 (3)
C1—C2—C3—C4	178.1 (2)	C9—C10—C11—C12	177.3 (2)
C2—C3—C4—C5	−0.5 (4)	N1—C10—C11—C12	−1.3 (3)
C3—C4—C5—C6	1.3 (3)	C10—C11—C12—C13	1.0 (3)
C3—C4—C5—C8	−176.7 (2)	C10—C11—C12—C16	−178.23 (18)
C4—C5—C6—C7	−0.9 (4)	C11—C12—C13—C14	0.0 (3)
C8—C5—C6—C7	177.0 (2)	C16—C12—C13—C14	179.22 (18)
C3—C2—C7—C6	1.2 (4)	C11—C12—C13—C15	−179.27 (19)
C1—C2—C7—C6	−177.6 (2)	C16—C12—C13—C15	−0.1 (3)
C5—C6—C7—C2	−0.4 (4)	C12—C13—C14—N1	−0.7 (3)
N1—N2—C8—C9	0.7 (2)	C15—C13—C14—N1	178.58 (18)
N1—N2—C8—C5	−178.00 (17)	N2—N1—C14—C13	−178.02 (18)
C4—C5—C8—N2	166.40 (19)	C10—N1—C14—C13	0.5 (3)
C6—C5—C8—N2	−11.4 (3)	C17—O2—C16—O3	−1.8 (3)
C4—C5—C8—C9	−12.0 (3)	C17—O2—C16—C12	177.15 (17)
C6—C5—C8—C9	170.1 (2)	C11—C12—C16—O3	176.6 (2)
N2—C8—C9—C10	−0.6 (2)	C13—C12—C16—O3	−2.6 (3)
C5—C8—C9—C10	177.97 (19)	C11—C12—C16—O2	−2.3 (3)
C8—C9—C10—N1	0.2 (2)	C13—C12—C16—O2	178.51 (17)
C8—C9—C10—C11	−178.5 (2)	C16—O2—C17—C18	−175.41 (18)
N2—N1—C10—C9	0.2 (2)		

### Hydrogen-bond geometry (Å, °)

**Table:**

*D*—H···*A*	*D*—H	H···*A*	*D*···*A*	*D*—H···*A*
C9—H9A···O3^i^	0.93	2.42	3.339 (3)	170

Symmetry codes: (i) *x*+1, −*y*+3/2, *z*+1/2.
